# Double-positive expression of high-mobility group box 1 and vascular endothelial growth factor C indicates a poorer prognosis in gastric cancer patients

**DOI:** 10.1186/1477-7819-11-161

**Published:** 2013-07-18

**Authors:** Weiling He, Bing Tang, Dongjie Yang, Yuhuang Li, Wu Song, Tuckyun Cheang, Xinlin Chen, Yin Li, Lianzhou Chen, Wenhua Zhan, Wen Li, Yulong He

**Affiliations:** 1 Department of Gastrointestinal and Pancreatic Surgery, The first Affiliated Hospital of Sun Yat-Sen University, Guangzhou 510080, China; 2 Institute of Clinical and Translational Research, Department of Histology and Embryology, Zhongshan School of Medicine, Sun Yat-Sen University, Guangzhou 510080, China; 3 Department of Biomedical Statistics, College of Fundamental Medical Science, Guangzhou University of Chinese Medicine, Guangzhou 510006, China; 4 Department of Surgical Laboratory, The first Affiliated Hospital of Sun Yat-Sen University, Guangzhou 510080, China

**Keywords:** High-mobility group box 1 protein, VEGF-C, Gastric cancer, Tissue microarray, Prognosis

## Abstract

**Background:**

Although many studies have indicated that high-mobility group box 1 protein (HMGB1) is associated with oncogenesis and a worse prognosis, the prognostic value of HMGB1 in gastric cancer (GC) remains unclear. In the present work, we aimed to evaluate the role of HMGB1 in GC and examined whether aberrant expression of both HMGB1 and vascular endothelial growth factor C (VEGF-C) increased the malignant potential of GC.

**Methods:**

A total of 166 GC patients and 32 normal subjects were enrolled. HMGB1 and VEGF-C expression was detected by tissue microarrays (TMAs) and immunohistochemical staining. The correlation between HMGB1 and VEGF-C expression and their relationships with clinicopathological GC variables were examined. Univariate and multivariate analyses were performed using the Cox proportional hazard model to predict the factors related to the patients‘ overall survival rates.

**Results:**

HMGB1 and VEGF-C expression were observed in 81 (48.80%) and 88 (53.01%) tumors, respectively, significantly higher than the rates among the corresponding controls. In addition, HMGB1 and VEGF-C expression were positively correlated (R^2^ = 0.972). HMGB1 expression was also closely associated with tumor size, pT stage, nodal status, metastasis status, TNM stage, and poor prognosis. Multivariate survival analysis indicated that patients with HMGB1 and VEGF-C coexpression had the worst prognoses and survival rates (hazard ratio, 2.78; log rank *P*<0.001).

**Conclusions:**

HMGB1 is commonly expressed in GC. Combined evaluation of HMGB1 and VEGF-C may serve as a valuable independent prognostic factor for GC patients.

## Background

Gastric cancer (GC) is a highly malignant disease with a poor prognosis, and is the second most common cause of cancer-related death worldwide. GC tends to be associated with hematogenous metastasis, peritoneal dissemination, and lymph node metastasis. For patients with advanced stage GC, the 5-year survival rate is approximately 20%. Multiple steps and factors involved in tumorigenesis, tumor invasion, and metastasis of GC influence disease prognosis, particularly metastatic dissemination of the primary tumor. However, the mechanisms of metastasis remain elusive. The identification of a predictive marker to evaluate the behavior of tumor development and metastasis would be valuable in clinical practice.

High-mobility group box 1 protein (HMGB1), also known as amphoterin, is a relatively small, versatile protein of 215 amino acid residues. Originally characterized as a non-histone nuclear-DNA-binding protein, HMGB1 can stabilize the structure and function of chromatin and regulate gene transcription [1,2]. It is also present in the cytoplasm and stroma cells and can be associated with tumor formation, proliferation, progression, metastasis, and chemotherapeutic response [3]. Overexpression and cytoplasmic localization of HMGB1 is associated with the proliferation and metastasis of many tumor types, particularly in conjunction with the receptor for advanced glycation end products (RAGEs) [4,5]. Increased expression of HMGB1 occurs in many solid tumors, including breast cancer [6], gastric cancer [7,8], colon cancer [9,10], and nasopharyngeal carcinoma [11]. Its aberrant expression is usually associated with the proliferation and metastasis of tumors and a worse prognosis [8-12]. Although serum levels of HMGB1 have been shown to be a useful marker to predict poor prognosis in GC [13], another study reported that overexpression of HMGB1 was positively correlated with cancer-free survival of resectable gastric adenocarcinomas [7]. Therefore, detailed studies with consecutive and extensive sample numbers are needed to confirm the clinical value of HMGB1 as a prognostic factor in GC.

Vascular endothelial growth factor C (VEGF-C) is a specific growth factor that targets the lymphatic system and plays a critical role in tumor growth and metastasis to lymph nodes and distant organs through the formation of new vessels in various malignancies, including GC [14-16]. HMGB1 was previously reported to enhance the invasion ability of tumor cells through a VEGF-C-related pathway [17], and HMGB1 promotes lymphangiogenesis in human lymphatic endothelial cells [18]. Thus, we have attempted to determine if HMGB1 promotes tumor metastasis by upregulating the expression of VEGF-C or if each protein exerts its function independently. Because simultaneous expression of HMGB1 and VEGF-C has not been examined in a well-characterized series of GCs with long-term follow-up, the present study serves to re-evaluate the application of HMGB1 and VEGF-C expression as a prognostic predictor in GC.

## Methods

### Patients

A total of 166 consecutive patients with gastric cancer who underwent gastrectomy at the Department of Gastrointestinal and Pancreatic Surgery of the First Affiliated Hospital of Sun Yat-Sen University from 2003 to 2005 were included in the present study. Patients who received chemotherapy, radiotherapy, and/or biotherapy before surgery were excluded from the study. The pathologic stage of the disease was determined according to the American Joint Committee on Cancer (AJCC) TNM staging system. All treatment plans were designed according to the latest National Comprehensive Cancer Network (NCCN) guidelines for GC. In the group, 139 patients received radical operations with neither gross nor microscopic evidence of residual disease, and these patients were included in the survival and prognostic analyses. Overall survival was defined as the length of time from surgery to death or to the last follow-up in the chosen patients. Normal subjects included non-gastric cancer cases excluded by histopathology under a gastroscope. The use of tissue samples for tissue microarray (TMA) analyses and clinical data were approved by the Medical Ethics Committee of the First Affiliated Hospital of Sun Yat-Sen University and the patients.

### Tissue array construction and immunohistochemical staining

Cores of 1 mm were removed from formalin-fixed paraffin-embedded samples of GC and adjacent normal gastric tissues. For all of the arrays, three cores from different areas of the tumor were removed for each case and were placed into a new blank recipient paraffin block as previously described by Hsu *et al*. [19], and 4-μm thick sections were taken for immunohistochemistry. Immunohistochemical staining for HMGB1 and VEGF-C was performed according to protocols available at http://www.abcam.com/index.html?pageconfig=popular_protocols. Samples were subsequently incubated with anti-HMGB1 (1:100, Santa Cruz Biotechnology, Santa Cruz, CA, USA) or anti-VEGF-C antibody (1:100, Santa Cruz) and visualized with an Envision Chem Detection Kit (Dako Cytomation, Carpinteria, CA, USA).

### Histological and immunohistochemical assessment

All slides were coded and evaluated blindly by two independent experienced pathologists. The final values of the positive tumor cells were assessed as the mean value of the immunoreactivity of five randomly selected areas of each section for correlation and confirmation of the tissue analysis. VEGF-C immunoreactivity was defined as a cytoplasmic staining pattern and HMGB1 as a nuclear staining pattern with or without cytoplasmic staining. Scores of the positive ratios were evaluated according to a previous report [7] as follows: score 0, <5%; score 1, 5% to 24%; score 2, 25% to 49%; score 3, 50%to 100%. For the purpose of statistical analysis, we combined samples with scores of 0 and 1 to represent negative expression and scores of 2 and 3 to represent positive expression.

### Cell lines

The immortalized human gastric epithelial mucosa cell line GES-1 and three human GC cell lines (MGC803, AGS and HGC27) were obtained directly from the Committee of Type Culture Collection of the Chinese Academy of Sciences (Shanghai, China) and passaged in our laboratory for less than 6 months after receipt. GES-1 was cultured in Dulbecco’s Modified Eagle Medium (DMEM; Gibco, Carlsbad, CA, USA), and GC cell lines were cultured in RPMI 1640 medium (Gibco, Carlsbad, CA, USA) supplemented with 10% fetal calf serum (HyClone, Logan, UT, USA) at 37°C in a humidified atmosphere of 5% CO_2_.

### Preparation of conditioned medium

To determine the effect of HMGB1 on VEGF-C expression, cell culture media was analyzed with a VEGF-C ELISA Kit (R&D Systems, Minneapolis, MN, USA). Briefly, 1×10^4^ cultured cells were incubated with human recombinant HMGB1 (hrHMGB1, Sigma, St. Louis, MO, USA) for 24 h. Then, the cultured medium was filtered with a 0.2-μm push filter (Millipore, MA, USA) and analyzed by ELISA [17,20].

### Statistical analysis

Statistical analysis was performed using the statistical software package SPSS 16.0 (SPSS Inc., Chicago, IL, USA). The associations between HMGB1 and VEGF-C expression and clinicopathological parameters were assessed. To identify the independent factors that may be significantly related to patient prognosis, univariate analysis and multivariate survival analyses were performed using a Cox proportional hazard model. The survival rates of patients with different HMGB1 or VEGF-C expression status were analyzed by the Kaplan-Meier method, and the survival curves were stratified according to HMGB1 and VEGF-C expression status by the log-rank test. All tests were two-sided, and *P *values of less than 0.05 were considered statistically significant.

## Results

### Expression of HMGB1 and VEGF-C proteins in GC

**Figure 1:**
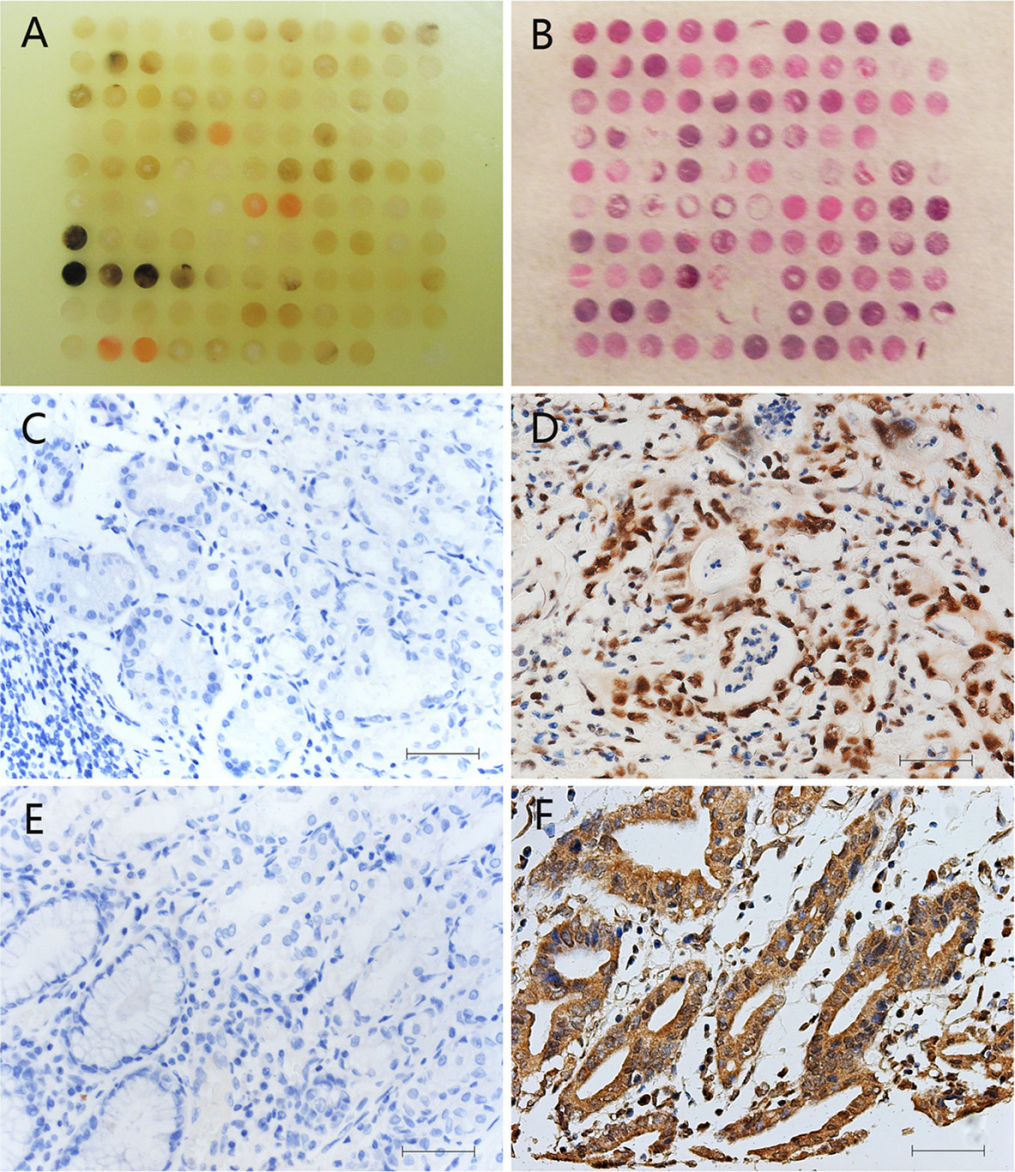
**Immunohistochemical labeling of vascular endothelial growth factor C (VEGF**-**C) and high-mobility group box 1 (HMGB1) in primary gastric cancer (GC) and normal gastric tissue.** Tissue microarrays of GC and non-gastric carcinoma tissue are shown **(A,B)**. HMGB1 was significantly increased in GC tissues. HMGB1 was mainly localized to the nucleus but,when compared to the normal gastric tissue **(C)**, was effusive to the cytoplasm and stroma in some cases **(D)**. The cytoplasmic expression of VEGF-C,when compared to the negative corresponding samples **(E)**, yielded similar results **(F)**. Bar, 50 μm.

HMGB1 protein expression was evaluated by immunohistochemical staining. As a non-histone DNA binding protein, the expression of HMGB1 protein was mainly localized to the nucleus but was also effusive in the cytoplasm and the stroma in some cases (Figure 1D). Positive staining was detected in 81/166 (48.80%) GC cells and 5/32 (15.6%) in non-cancerous cells. The difference in these staining patterns was statistically significant (*P*<0.05; Figure 1C). VEGF-C was observed almost exclusively in the cytoplasm of gastric tumor cells, as previously reported [17,20] (Figure 1F). The positive rate of VEGF-C staining in the primary tumor was 53.01%, and no positive staining was observed in non-cancerous gastric tissues (*P*<0.05, Figure 1E).

### Correlation of HMGB1 and VEGF-C expression with clinicopathological characteristics

**Figure 2:**
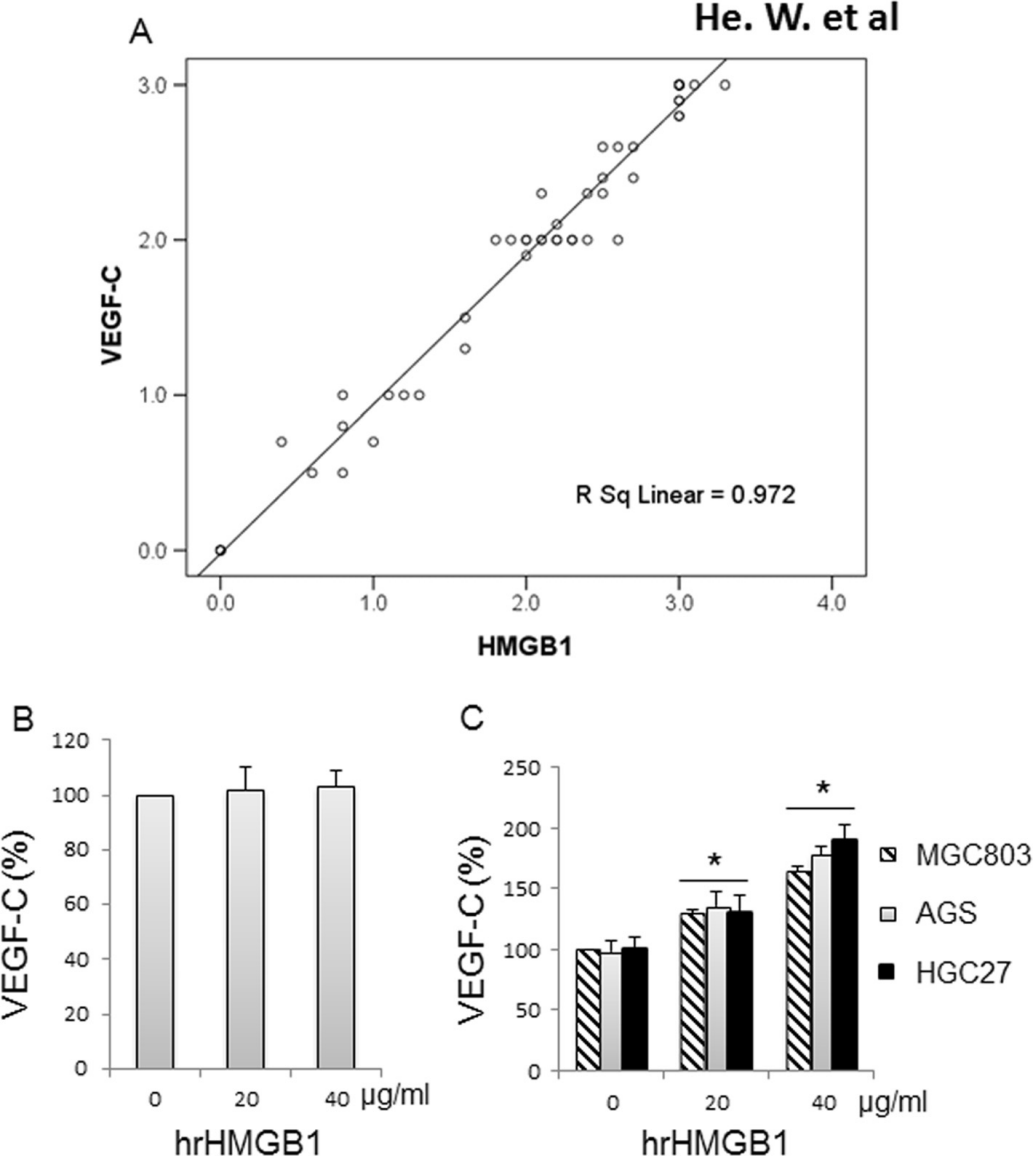
**Correlation between vascular endothelial growth factor C (VEGF-C) and high-mobility group box 1 (HMGB1).** A correlation coefficient revealed that VEGF-C expression was highly correlated with HMGB1 in gastric cancer (GC) **(A)**. *In vitro*, cells were incubated with human recombinant HMGB1 (hrHMGB1) for 24 h, and the cultured medium was then used to determine the VEGF-C concentration. VEGF-C secretion was not significantly upregulated by HMGB1 in a dose-dependent manner in GES-1 cells **(B)**,but was in GC cells **(C)**.

The relationship between HMGB1, VEGF-C expression, and various clinicopathological features (tumor size, pT stage, distant metastasis, nodal status, and TNM stage) was analyzed (Table 1). The expression of HMGB1 was significantly correlated with tumor size, pT stage, lymph node metastasis, and TNM stage (Table 1) but not with age, gender, pathological type, or tumor differentiation. Meanwhile, VEGF-C expression displayed associations with clinicopathological characteristics similar to those of HMGB1 (Table 1). In addition, statistical analysis demonstrated that there was a significant correlation between HMGB1 and VEGF-C expression (Figure 2A, R^2^ = 0.972, *P*<0.01).

**Table 1:** Association between the expression of vascular endothelial growth factor C (VEGF-C) and high-mobility group box 1 (HMGB1) with clinicopathological characteristics of patients with gastric cancer

**Clinicopathological characteristics**	**No**.	**HMGB1**		**Ratio (%)**	***P *value**	**VEGF**-**C**		**Ratio (%)**	***P *value**
		**Positive**	**Negative**			**Positive**	**Negative**		
Total	166	81	85	48.80		88	78	53.01	
Age at surgery:									
<60	84	45	39	53.57	0.213	47	37	55.95	0.442
≥60	82	36	46	43.90		41	41	50.00	
Gender:									
Male	113	59	54	52.21	0.198	59	54	55.66	0.763
Female	53	22	31	41.51		29	24	59.18	
Histological type:									
Adenocarcinoma	135	62	73	45.93	0.123	69	66	51.11	0.306
Other	31	19	12	61.29		19	12	61.29	
Tumor size:									
<4 cm	83	30	53	36.14	0.001	32	51	38.55	<0.001
≥4 cm	83	51	32	61.45		56	27	67.47	
Borrmann type:									
I+II	35	16	19	45.71	0.681	17	18	48.57	0.553
III+IV	131	65	66	49.62		71	60	54.20	
Differentiation:									
Low	113	56	57	49.56	0.774	58	55	51.33	0.525
Moderate andhigh	53	25	28	43.24		30	23	54.05	
pT stage:									
pT1 to pT2	34	9	25	26.47	0.003	10	24	29.41	0.002
pT3 to pT4	132	72	60	54.55		78	54	59.09	
Distant metastasis:									
M0	122	53	69	43.44	0.022	57	65	46.72	0.007
M1	44	28	16	63.64		31	13	70.45	
TNM stage:									
Stage I+II	61	17	44	27.87	<0.001	17	44	27.87	<0.001
Stage III+IV	105	64	41	60.95		71	34	67.62	
Nodal status:									
pN0	63	19	44	30.16	<0.001	21	42	33.33	<0.001
pN1 to pN3	103	62	41	60.19		67	36	65.05	

### HMGB1 upregulates VEGF-C secretion in GC cell lines

To further evaluate the effects of HMGB1 on VEGF-C, we treated GES-1 and GC cells with hrHMGB1. VEGF-C secretion was remarkably increased by incubation with hrHMGB1 in a dose-dependent manner in GC cells (Figure 2C) but not in GES-1 cells (Figure 2B). These data support the correlation between HMGB1 and VEGF-C *in vivo*.

### Prognostic value of HMGB1 and VEGF-C expression in GC patients

**Figure 3:**
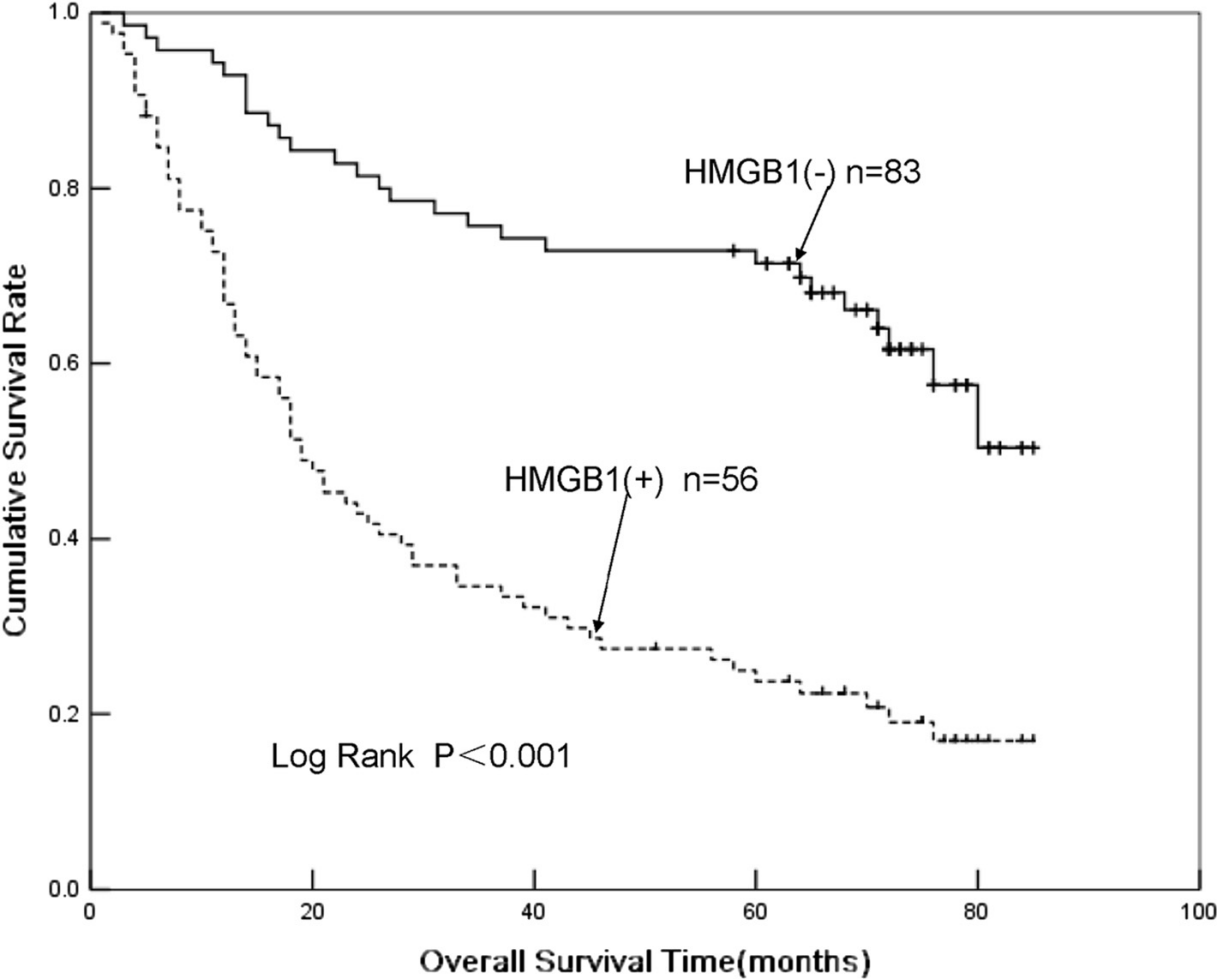
**Kaplan-Meier plots for the cumulative 5-year survival of patients with gastric cancer (GC) after radical resection, stratified according to high-mobility group box 1 (HMGB1)expression status.** Patients with tumors strongly positive for HMGB1 had significantly poorer prognosis than other patients (*P*<0.001, log-rank test).

**Figure 4:**
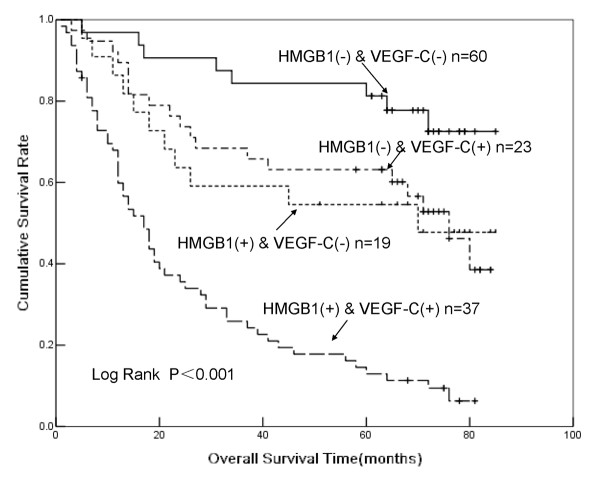
**Kaplan-Meier plots for the cumulative 5-year survival of patients with gastric cancer (GC) after radical resection, stratified according to vascular endothelial growth factor C (VEGF-C) and high-mobility group box 1 (HMGB1) expression status.** Patients who had tumors with combined HMGB1 and VEGF-C expression had significantly poorer prognosis than other patients (*P*<0.001, log-rank test).

Because HMGB1 and VEGF-C expression were both significantly associated with tumor invasion and metastasis in GC (Table 1), we further investigated whether these two immunohistochemical markers could be used as prognostic predictors for GC. A total of 139 patients who received radical operations with standard D^2^ or extended lymph node dissection were included in the prognostic analysis. Univariate analysis revealed that tumor size, pT stage, lymph node metastasis, TNM stage, histological differentiation, HMGB1 expression, and VEGF-C expression were all significantly associated with poor survival rates (data not shown). As expected, patients with positive HMGB1 staining (+) had a much poorer prognosis than those with negative HMGB1 (−) staining (Figure 3). Furthermore, Cox’s multivariate analysis revealed that lymph node metastasis, TNM stage, HMGB1 expression, VEGF-C expression, and combined HMGB1/VEGF-C expression were significant predictors of survival (Table 2). Survival curves plotted according to the Kaplan-Meier method revealed that patients with combined HMGB1/VEGF-C negative expression had the best survival rates, whereas patients with positive expression of both markers had the worst survival rates. Those with abnormal expression of either VEGF-C or HMGB1 had an intermediate survival rate (Figure 4).

**Table 2:** Cox regression survival analysis of factors predicting survival time of patients with gastric cancer

**Clinicopathological characteristics**	**Hazard ratio**	**95.0% CI for Exp (B)**	***P *value**	
			**Lower**	**Upper**
Borrmann:				
I and II	1			
III and IV	0.78	0.21	2.85	0.7072
Histologic differentiation:				
Well and moderate	1			
Poorly	1.26	0.67	2.38	0.4756
Tumor size:				
<4 cm	1			
≥4 cm	1.17	0.63	2.18	0.6277
pT stage:				
pT1 and pT2	1			
pT3 and pT4	1.99	0.60	6.60	0.2595
Lymph node metastasis:				
Absence	1			
Presence	2.58	1.01	5.06	0.0480
TNM stage:				
I, II	1			
III, IV	2.70	1.06	7.03	0.0296
High-mobility group box 1 (HMGB1)expression:				
Negative	1			
Positive	2.66	1.04	6.32	0.0370
Vascular endothelial growth factor C (VEGF-C) expression:				
Negative	1			
Positive	2.62	1.03	6.83	0.0464
HMGB1 and VEGF-C				
Others^a^	1			
Combined HMGB1and VEGF-C expression				
	2.78	1.21	6.40	0.0162

^a^Others were HMGB1 negative and VEGF-C negative, HMGB1 negative and VEGF-C positive, and HMGB1 positive and VEGF-C negative.

## Discussion

In the present study, we provide the first evidence that coexpression of HMGB1 and VEGF-C is associated with aggressive biological behavior and poor prognosis in a large series of human GCs and is a valuable prognostic marker.

HMGB1, the focus of many recent cancer studies, plays a critical role in cancer development, progression, and metastasis through its pro-angiogenic and pro-lymphangiogenic functions [18]. Consistent with other studies, the HMGB1 protein was highly expressed in GC samples. We also observed that the positive staining signal was mainly localized to the nucleus in GC but was also found in the cytoplasm and stroma, in accordance with its biological function. Hypoxia results in necrotic or damage-induced cell death within the tumor when the growing tumor exceeds the capacity of the existing vasculature. HMGB1 not only activates vascular endothelial cell proliferation and neovascularization through the HMGB1-RAGE pathway, it can also stimulate inflammation [21]. Damaged or necrotic cells can actively secrete or passively release HMGB1 into the extracellular milieu. The constant release of the pro-inflammatory cytokine HMGB1 from necrotic tumor cells creates a microenvironment similar to chronic inflammation and contributes to the development of epithelial malignancies [22]. Furthermore, the rate of HMGB1 expression (48.80% of 166) is within the range of previously reported data [8-12]. Moreover, we observed that HMGB1 expression was closely correlated with TNM stage, nodal status, and survival rate but not with age, gender, or pathological type, as reported previously [8-12]. Controversially, we did not observe any significant differences in HMGB1 expression between well-differentiated tumors and poorly differentiated ones, similar to the results obtained by Hao *et al*., in which no correlation between HMGB1 expression and tumor differentiation was observed [23]. Further studies of HMGB1 expression in GC and its function are needed.

As expected, the correlation of HMGB1 expression with GC prognosis is in accordance with most of the findings of previous studies. HMGB1 expression has been reported to be significantly associated with tumor invasion, lymph node metastasis, distant metastasis, and Duke’s stage and inversely associated with overall survival in human colorectal carcinoma [10]. HMGB1 has been shown to be an independent prognostic factor for patients with squamouscell carcinoma of the head and neck [12], and its overexpression also plays a role in the progression of nasopharyngeal carcinoma (NPC) and is correlated with a poor clinical outcome [11]. Serum HMGB1 is closely associated with the clinical and pathological features of GC and appears to be a useful serological biomarker for early diagnosis as well as the evaluation of tumorigenesis, stage, and prognosis in GC [13]. Regarding the potential mechanism, Ohmori *et al*. demonstrated that HMGB1 enhances the proliferation, motility, invasion, and survival of cancer cells, induces apoptosis of macrophages, and suppresses the host anti-cancer immune system [24].

However, several studies differ from our conclusion. Bao *et al*. concluded that there was no significant association between HMGB1 expression and invasion depth, tumor stage, and lymph node metastases. Curiously, their results suggested that overexpression of HMGB1 was positively correlated with patient prognosis after curative resection and adjuvant chemotherapy [7]. Akaike *et al*. reported that the prognosis of the low HMGB1 group was significantly poorer than that of the high HMGB1 group in GC [8]. Similarly, Jiao *et al*. proposed that HMGB1 could function as a tumor suppressor and radiosensitizer in breast cancer [25] because oxidized HMGB1 increased the cytotoxicity of these agents and induced apoptosis via the mitochondrial pathway or the caspase-9/-3 intrinsic pathway [26]. Furthermore, HMGB1 plays a critical role in tumor immunology. The interaction of the HMGB1 protein released from dying tumor cells with Toll-like receptor 4 on dendritic cells is required for the crosspresentation of tumor antigens and the promotion of tumor-specific cytotoxic Tcell responses [27,28], which are selectively involved in the crosspriming of anti-tumor T lymphocytes *in vivo*[29,30]. This discordance may be due to the various tumor types, intricate microenvironments, and different responses to follow-up treatment in these studies. Further study of the underlying mechanism of this discordance is a worthwhile and beneficial avenue to elucidate the precise role of HMGB1 in tumorigenesis.

VEGF-C is a classic specific growth factor of the lymphatic system that is also known to play several roles in tumor growth and metastasis to lymph nodes and distant organs. VEGF-C may induce angiogenesis and lymphangiogenesis in malignant tumors [31,32]. Metastasis to regional lymph nodes and distant organs through the expression of VEGF-C has been identified in several cancers such as colon cancer, prostate cancer, and gastric cancer [16,33-38]. Similarly, in our study, expression of VEGF-C was significantly associated with TNM stage, nodal status, and poorer overall survival [39-45].

Whether HMGB1 and VEGF-C act independently or cooperatively to increase the malignant potential of GC is not clear. We verified the high correlation between these two markers in GC tissues and found that HMGB1 could increase VEGF-C secretion in GC cells (Figure 2). We also demonstrated that HMGB1 could upregulate VEGF-C secretion in GC cell lines, suggesting that HMGB1 may enhance the invasion ability of tumor cells, at least in part, through VEGF-C-related pathways [17,20]. As expected, Kaplan-Meier survival analysis indicated that patients with expression of both markers had the poorest prognoses when compared to all other groups. Correspondingly, individuals negative for both markers displayed the longest survival, while patients expressing a single marker had intermediate survival rates (Figure 4). Therefore, assessment of HMGB1 and VEGF-C in preoperative biopsies may assist in the stratification of GC patients according to different optimized treatment protocols, such as adjuvant radical or chemotherapy.

The application of molecular diagnosis can be beneficial for the earlier and more accurate identification of cancer prognosis or grade of malignancy, thus offering rapid and efficient therapy [46]. Accumulating evidence suggests that HMGB1 and VEGF-C are useful adjunct markers for traditional prognostication indices [9-12,39-45]. In this study, we further confirmed the predictive value of the molecular approach and verified the benefit by simultaneously evaluating the expression of HMGB1 and VEGF-C (hazard ratio 2.78, *P* = 0.0162). Nevertheless, the mechanism by which coexpression of HMGB1 and VEGF-C promotes GC progress and metastasis needs to be further investigated.

## Conclusions

HMGB1 expression is dysregulated in GC and is significantly correlated with several clinicopathological characteristics, as is VEGF-C expression. The combined evaluation of HMGB1 and VEGF-C expression in GC tissues facilitates the prediction of clinical prognosis for patients with GCwho are surgically treated. Double-positive expression of HMGB1 and VEGF-C is an independent prognostic factor for survival in patients with GC and a distinguishing factor for the subgroup of patients with the worst prognosis.

## Competing interests

The authors declared that they have no competing interests.

## Authors’ contributions

WH and BT drafted the manuscript and participated in all other parts of the work. DY performed the immunoassays. TC and YL performed the molecular studies. WS participated in the design of the study. XC, YL, and LC performed the statistical analyses. WZ, WL, and YH conceived the study, participated in its design and coordination, and helped to draft the manuscript. All authors read and approved the final manuscript.
